# Access to Information, and Concerns, Myths and Truths about Food Safety during the COVID-19 Pandemic: An Overview of the Portuguese Population

**DOI:** 10.3390/foods12142802

**Published:** 2023-07-24

**Authors:** Marcela Lemos, Rui Leandro Maia, Paula Teixeira

**Affiliations:** 1 Universidade Católica Portuguesa, CBQF—Centro de Biotecnologia e Química Fina—Laboratório Associado, Escola Superior de Biotecnologia, Rua Diogo Botelho 1327, 4169-005 Porto, Portugal; marcelaclemos@gmail.com; 2 CITCEM—Centro de Investigação Transdisciplinar «Cultura, Espaço e Memória», Faculdade de Letras do Porto, Universidade do Porto, 4150-564 Porto, Portugal; 3 FP I3ID—Institute for Research, Innovation and Development Fernando Pessoa Foundation, Universidade Fernando Pessoa, 4249-004 Porto, Portugal

**Keywords:** communication, coronavirus pandemic, consumption habits, food safety, hand washing and disinfection

## Abstract

**Simple Summary:**

The COVID-19 pandemic raised questions and concerns about the possibility of the virus being transmitted through food, as the virus was found in sewage, shrimps and packages of frozen food. Due to the emerging need for information on SARS-CoV-2 as a novel virus, the media played an important role in disseminating information related to the hypothesis of food-borne transmission of SARS-CoV-2, which could have led to different public reactions related to food consumption and hygiene, food safety, and food supply chains. Identifying and understanding the main doubts and concerns about food hygiene and safety raised by the Portuguese population during the first wave of COVID-19 is important in order to understand whether these issues have influenced their practices and what lessons can be learnt for food safety and hygiene education. It was observed that the main concern expressed during this period was related to food handling due to the risk of SARS-CoV-2 transmission (41.6%), and television was the main source of information used to clarify these doubts (32.9%).

**Abstract:**

The COVID-19 pandemic raised questions and concerns about the possibility of the virus being transmitted through food, as the virus was found in sewage, shrimps and packages of frozen food. During the first wave of COVID-19, concerns about the transmission of SARS-CoV-2 through food arose. As the number of cases began to increase rapidly, so did the availability of information regarding the virus and ways to prevent infection. A significant portion of this information was disseminated by the media and the general public. Identifying and understanding the main doubts and concerns about food hygiene and safety raised by the Portuguese population during the first wave of COVID-19 is important in order to understand whether these issues have influenced their practices and what lessons can be learnt for food safety and hygiene education. The aims of this work were (1) to understand the doubts and concerns of the Portuguese population regarding food safety and hygiene during the first wave of COVID-19, and how these issues were clarified, (2) to analyze the population’s opinion on food/hygiene myths and truths related to the transmission and prevention of the infection, and (3) to understand how the first wave of COVID-19 may have influenced the population’s practices linked to food handling and consumption. The main doubts of the respondents were related to food handling (41.6%) and the possibility of transmission of COVID-19 through food (17%). Television was the main source of information used to clarify these doubts (32.9%), followed by a guideline issued by the Directorate-General of Health (30.7%). However, most respondents (50.9%) said that they had only found answers to some of their questions. Most respondents reported washing and disinfecting hands before (85% and 63.4%, respectively) and after (73.8% and 57.3%, respectively) the handling and organization of food purchases. Most respondents did not believe the myths about COVID-19 and food safety, but this depended on their level of education. Some practices may have changed as a result of the pandemic, particularly with regard to washing and disinfecting hands and food, as well as kitchen hygiene.

## 1. Introduction

The COVID-19 pandemic, caused by the SARS-CoV-2 virus, had a direct impact on lifestyles and habits worldwide, including behaviors related to food handling and consumption. As it was a new virus strain, little was known about its virulence, mode of transmission, viability in food, and prevention.

Even though a rapid increase in SARS-CoV-2 infections was observed worldwide [1,2,3,4], and COVID-19 was classified as an airborne disease [5,6], the possibility of foodborne transmission was still considered.

The first cases of COVID-19 were reported in a wet market in Wuhan, China, where wild animals and seafood were sold. This led to the hypothesis that the disease could be zoonotic, i.e., transmitted from animals to humans through direct contact or consumption [7,8,9,10]. This hypothesis was reinforced by messages circulating on the Internet linking the consumption of bats to the transmission of the disease, and by reports of the detection of SARS-CoV-2 in frozen foods, such as shrimps from Ecuador, chicken wings from Brazil and salmon in China [11,12,13,14,15,16,17]. Contaminated frozen salmon, even though the finding was in a small sample—six out of 3582 samples analyzed were positive for SARS-CoV-2 RNA—was considered as a possible source of a COVID-19 outbreak in Beijing [18]. Feng et al. [19] contaminated pork, beef and salmon meat samples with SARS-CoV-2 and evaluated its stability on meat surfaces during storage at −20 °C compared to 4 °C. The virus was recovered several hours to days after infection. The viral isolation rate depended on the type of sample, the level of inoculation and was higher when samples were stored at −20 °C than at 4 °C. This suggests that the virus may remain stable during food transport and packaging, potentially leading to transmission during food handling and consumption. Other studies support this hypothesis. Jung et al. [20] found that, although the virus was not viable at room temperature, its half-life increased to 24–46 h and 100 h in chicken, salmon and lettuce when stored at 4 °C or −40 °C, respectively. Dai et al. [21] observed the stability and infective potential of SARS-CoV-2 in salmon at 4 °C. Chin et al. [22] confirmed its stability at 4 °C in salmon, with decreased infectious capacity after 14 days. Fukuta et al. [23] detected SARS-CoV-2 in milk, fruit juice and alcoholic beverages for up to 77 days after inoculation, but its infectious potential declined over time. Jia et al. [24] demonstrated the infectiousness of SARS-CoV-2 in various meats for up to 21 days, while certain foods containing food additives, such as salami, showed antiviral effects. It was also observed that the levels of SARS-CoV-2 in spinach and apple skin remained constant for 24 h, and that mushrooms showed antiviral activity one hour after inoculation due to the antioxidants naturally present in these foods. Dhakal et al. [25] observed virus recovery in chicken and salmon after 24 h at 4 °C. Rafieepoor et al. [26] detected SARS-CoV-2 in samples from food production and a retail chain in Tehran, including agricultural water and vegetables from local markets. Oakenfull and Wilson [27] found a minimal risk of infection from consuming eggs, poultry, or fish, or contact with their packaging.

The possibility of foodborne transmission was nonetheless supported by reports of gastrointestinal symptoms [28,29,30,31,32,33]. There has also been evidence of SARS-CoV-2 in sewage and wastewater in the Netherlands, Finland, Liechtenstein and Spain [34,35,36,37,38], and anal swabs have been found to have been positive for SARS-CoV-2 for longer than have nasal swabs [38,39,40,41], even though further studies aiming to identify the viability of SARS-CoV-2 in feces are needed to confirm fecal–oral transmission.

Despite these studies, no cases of illness have been reported as a result of the consumption of foods. However, food hygiene is crucial, particularly for raw products, as viable virus can be found on their surfaces and studies are still needed to understand whether eating them can lead to illness, as is the case with norovirus, for example. The Food and Environment Reporting Network (FERN) [42] monitored COVID-19 cases in food processing facilities, with over 90,000 infections recorded. These findings suggest that food handling processing environments may represent an increased risk of SARS-CoV-2 infection due to the handling of potentially contaminated food [42,43].

Due to growing concerns about the transmission of SARS-CoV-2 through food, there was also a concern regarding the possibility of infection through contact with packaging of exposed products in supermarkets, where crowded environments increase the risk of droplet spread [5,6,44,45,46]. Doremalen et al. [47] found viable virus in aerosols for three hours, with a reduced infectiousness over time. The virus was viable for 72 h on plastic and 24 h on cardboard, with slight reductions in infectious potential. Liu et al. [41] observed a seven-day stability on plastic at room temperature, but viral load decreased over time. Kampf et al. [48] noted stability on plastic for two hours to nine days, but ethanol exposure (63–71%) for a minute effectively inactivated the virus. Because SARS-CoV-2 can remain stable at potentially infectious levels for days, the virus can potentially infect via contaminated surfaces, especially if brought into contact with mucous membranes. However, environmental factors and time can decrease its infectiousness, lowering the risk [49]. Jung et al. [50] found surface inactivation within 48 h at room temperature, and surface disinfection with 70% ethanol was able to inactivate the virus after 5 min.

Although there was no scientific evidence that SARS-CoV-2 could be transmitted through food [51], some researchers emphasized the importance, particularly at a time when vaccines were not yet available, of not taking this assumption for granted. They recommended implementing specific food safety measures to protect consumers (e.g., eating cooked or canned foods rather than unpackaged or uncovered foods, cleaning the surface of canned foods, disposing of packaging materials immediately after unpacking foods, and avoiding eating raw foods, especially meat products) [52,53].

Several studies in some countries in Asia, Europe and the Americas have shown a change in behavior of the population regarding what to eat, what to avoid and how to prepare food, as well as an increase in concerns and doubts regarding COVID-19 and food safety [51,52,53,54,55,56,57,58,59,60,61,62,63,64].

Surveys conducted by the International Food Information Council revealed that, in the US, before the pandemic, consumers’ main food safety concerns were foodborne illnesses caused by bacteria, chemicals in food and carcinogens in food. However, the emergence of COVID-19 brought attention to a new issue: the risk of contracting the virus through food handling and preparation; in 2020, this was added to the list of top food safety concerns [65]. Doubts about foodborne transmission, particularly about “recently bought grocery” products and concerns about the need to “always wash the hands”, were also expressed by the population, as reported in a previous study in Portugal [58].

Several studies in different countries have reported the intensification of hygiene practices during shopping and during handling of products, as well as in the home, to avoid contamination with coronaviruses, e.g., disinfection of surfaces such as shopping trolleys or baskets before use, careful removal of food packaging, wiping/disinfection of food packaging, and washing and disinfection of fruits and vegetables [59,60,61]. Handwashing and disinfection, including before and after handling food and food packaging, was probably one of the most widely practiced measures used by the general public during the pandemic [57,60,61,62,63]. While the primary motivation was to protect against SARS-CoV-2 [61], these practices still played a critical role in minimizing the risk of transmission of foodborne pathogens. People’s increased awareness of personal hygiene also led to significant changes in the food safety environment. For example, Jung et al. [62] reported that the pandemic influenced communal eating practices in South Korea, emphasizing the importance of individual portions of shared dishes and the use of personal plates to promote good hygiene.

The impact of COVID-19 on diet has revealed some controversies, with some positive shifts towards healthier choices (e.g., increased consumption of fruits, vegetables and nuts and decreased consumption of fast food and alcohol) [57,66,67], but also negative shifts towards comfort eating and decreased nutritional quality, contributing to an increase in the prevalence of obesity [68].

The sources of consumer information on COVID-19 and related protective measures, including food safety, and their credibility have been investigated in numerous studies. It has been shown that people generally relied on traditional media sources such as television, radio and newspapers to learn new information related to the pandemic [69]. However, this preference for traditional media appears to have varied by age, particularly in the use of television news and various forms of social media; older people tended to rely more on traditional media, while younger people were more likely to use various forms of social media platforms to find their news [70]. In general, the World Health Organization (WHO), health authorities and health professionals were considered to be the most reliable sources of information, and this is consistent across studies [71,72].

This study aimed to identify the main doubts and concerns related to food safety during the first wave of COVID-19 in Portugal and if/how these issues were addressed, as there is little information available on the communication carried out during the pandemic and how it may have influenced the habits of the population. The study also aimed to analyze the population’s opinion on food/hygiene myths and truths related to the transmission and prevention of the infection and how the first wave of COVID-19 pandemic in Portugal might have influenced their practices related to food handling and food consumption. These results can offer valuable insights that can be utilized to educate consumers on food safety and hygiene practices. Furthermore, the results can inform decision-making by authorities during the post-pandemic period and in the event of future epidemics or other emergency situations.

## 2. Materials and Methods

### 2.1. Survey Construction and Administration 

The construction of the survey began after informal discussions with consumers on various topics related to COVID-19 and food. The aim of these discussions was to identify sources of information available to the general population on hygiene and food safety, specifically in relation to the prevention of the spread of SARS-CoV-2 and COVID-19. In addition, publications on the subject and other sources of information were researched in the scientific literature and the grey literature. Based on the information gathered, a draft questionnaire was developed, tested and reviewed by 10 individuals with regard to the terms used, the types of questions to be included, all the possible answers, and the forms of analysis to be taken into account [73].

This resulted in the final survey (Supplementary Information), consisting of a set of 18 questions, intentionally organized into two classic groups, which allow, simultaneously, watertight and combined readings and interpretations [74,75]. One group refers to facts and occurrences, that is, it permits the description of sociodemographic profiles and experiences, including the characterization of dimensions that would likely influence practices, opinions, perceptions and understandings of consumers, such as gender, age, level of education, location and whether professionally active. The other group is based on self-descriptions of things understood, perceptions and opinions, severally organized in Likert scales adapted according to the nature of the questions, which oscillate between opposites, such as (a) “never” to “more”; (b) “never” to “always”; (c) “never” to “more than once a week”; and (d) “I agree” to “I have no opinion”.

The survey was launched online between June and October of 2020. The online format was adopted given the travel and contact restrictions during the pandemic, and also due to the greater availability of participants, using online platforms, to respond from their homes. Snowball sampling was used for the dissemination of the survey. This is a non-probabilistic sampling technique based on the possibility of adding participants until a reasonable number of responses are obtained within the time allotted for this purpose [76,77].

The survey, titled “Food safety during the pandemic”, was available in Portuguese and created using the Google Forms tool provided by Google; it consisted of four main parts:

1. Concerns and information about food safety during the first wave of COVID-19 pandemic;
2. Behaviors before and during the first wave of COVID-19 pandemic;
3. Myths and truths about COVID-19;
4. Respondent’s profile.

The responses were organized in a Microsoft Excel spreadsheet and coded in numbers to be statistically analyzed.

### 2.2. Data Analysis

Data analysis was carried out using IBM^®^ SPSS^®^ software (Statistics International Business Machines Corporation, Armonk, NY, USA) version 27.0, using frequency tables to evaluate the data obtained and describe the participants in the survey. Statistical analysis was performed with the same software, using the Chi-square test. The odds ratio was defined as 95% (*p* ≤ 0.05), and frequencies were presented as numbers (percentual). For statistically significant results (*p* ≤ 0.05), Cramer’s V was also considered to verify the degree of association between variables, in which a value close to 0 (zero) indicated an absence of association or a weaker association, and a value close to 1 (one) indicated a stronger association.

Since a Chi-square test was utilized, data was presented as “*n*”, which refers to absolute frequency, and “%”, which refers to relative frequency.

**Figure 1:**
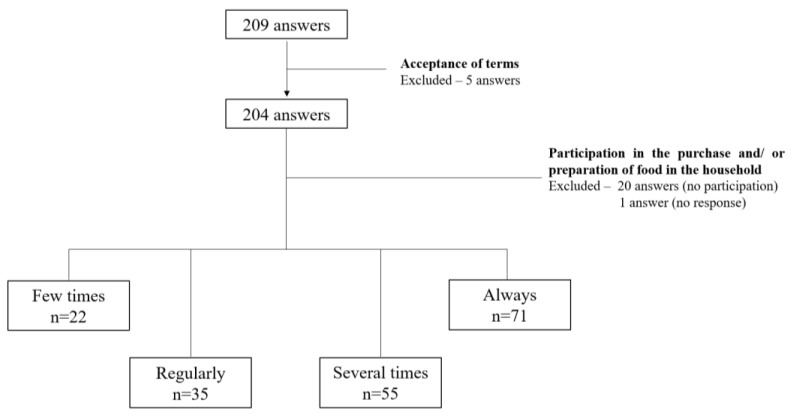
Representative scheme of the inclusion and exclusion criteria for the study.

Only the responses of people who agreed to the terms of the survey and who reported some involvement in purchasing and/or preparing food in their household were included in the statistical analysis. There were 209 responses to the survey, and 97.6% (*n* = 204) of the participants agreed to the terms of the survey. Regarding their participation in the purchase and/or preparation of food in the household, responses marked as “never” (*n* = 20) and no response (*n* = 1), were excluded, leaving 183 answers (Figure 1) for the study.

Questions to which there were no responses were also not considered for the statistical analysis, which was done randomly, with the overall rate of use of the survey standing at 87.6%, indicating that the disregard of these data did not impact the final analysis [78,79].

### 2.3. Sample Description

The sociodemographic profile of the respondents is presented in Table 1. Most of the participants were female (76.5%), had a bachelor’s degree or a technical or professional degree (40.4%), lived in Portugal (91.8%) and were employed (72.7%) (Table 1). People in employment—those who had a paid full-time or part-time job—participated more actively in this process (42.5%, *p* = 0.023, Cramer’s V = 0.229) (Table 2). Based on the evaluation of the sociodemographic profiles, the results were organized according to the most frequently occurring groups, since they represented a more significant part of the population studied.

## 3. Results and Discussion

The rapid transmission and spread of the novel SARS-CoV-2 virus, coupled with its high infectivity and significant morbidity and mortality, as well as the limited understanding of treatment and control methods [7,8,9,10], raised several questions about the virus and the disease it caused. This stimulated the scientific community to respond rapidly to these questions in order to understand the behavior of this virus and to identify ways to prevent infection and promote its treatment and cure.

For participants in this survey, the most important issue that arose during the COVID-19 pandemic was related to food handling (41.6% of responses), followed by doubts about the possibility of COVID-19 transmission through food (17%), and which foods should be avoided during the pandemic (9.1%). A total of 26.7% of the participants stated that they had no such doubts, a result particularly found among those with a bachelor’s, technical, or professional degree (31.1%). Gender may have influenced doubts during the COVID-19 pandemic, but the degree of association between the variables is small (Table 3).

At the same time as the concerns were raised, the media quickly disseminated information about the virus, including WHO messages and news of scientific advances [80,81,82]. In the present study, 32.9% of the respondents indicated that they had used television as the main means of communication for clarifying doubts and concerns related to the COVID-19 pandemic, followed by the guidelines on food safety during COVID-19 issued by the National Directorate-General of Health (DGS) and news websites, such as “Portal SAPO”, “Observador” and “Notícias ao Minuto” (30.7% and 9.8%, respectively) (Table 4). Contrary to what was reported by Lühnen et al. [69], age does not seem to have influenced the choices of the respondents. In general, this in agreement with previous data from the Special Eurobarometer on Food Safety in the EU (2019), indicating that television (69%), the Internet (excluding social media) (46%) and newspapers/magazines (38%) are the primary sources of food risk information in the EU. The lower frequency reported for printed newspapers is probably justified by people’s inclination to avoid going outside for shopping due to lockdown measures and their fear of contracting the virus [83].

Participants were also queried about their utilization of websites from various organizations that offered information, such as DGS, the Portuguese Economic and Food Safety authority (ASAE), the European Food Safety Authority (EFSA), and the Portuguese National Institute of Health (INSA), to seek additional information. A total of 35% of respondents stated they did not consult any website, while 59.6% said they had visited the DGS website (Table 5).

Information access and understanding vary across cultures. Reliance on social networks as an information source differs between countries. In a Spanish study [56], 37% of participants used social networks as their main communication source during the pandemic, compared to only 3.3% in this study. Brazil and the United States also have higher social networking engagement [60,84,85,86], enabling easy and rapid information access. However, this carries a high risk of misinformation, due to the lack of verification from official sources. Television, requiring rigorous source verification, is generally more reliable. Yet, it may be slower in disseminating information and not fully address all questions, as seen in our survey, where 51% of participants couldn’t find answers to all their questions (Table 6). This could lead people to seek answers from less reliable sources, increasing the spread of false information. For example, Verma et al. [87] found that 70 (out of 100) YouTube videos had incorrect produce-washing information. Bloggers’ videos had more incorrect information than those created by the government and other organizations, potentially leading to harmful consumer behavior. The videos shared during the pandemic were more likely to contain nonfactual information than those posted before the pandemic.

During the COVID-19 pandemic, an increase in fake news dissemination occurred, and this misinformation could have had health consequences and affected the effectiveness of official pandemic control measures. False messages spread on social networks promoted home remedies such as citrus fruits, ginger, garlic and medicinal plants (e.g., artemisia, eucalyptus) as preventive and symptomatic treatment options, despite the lack of scientific evidence [11,88,89]. Additionally, there were suggestions that ingesting bleach and other disinfectants or regularly consuming alcoholic beverages could prevent infection or hinder the virus’s multiplication [12,88,89,90,91,92,93]. In this study, there was generally a good distinction between myths and truths about COVID-19, with the majority of participants disagreeing with: “drinking alcohol can help prevent infection with the new coronavirus”, “drinking water with a few drops of bleach can help prevent infection” and “eating citrus fruits can help prevent infection with the new coronavirus” (Table 7).

In Portugal, the most discussed topic on “Polígrafo” during the pandemic was COVID-19 [94]. “Polígrafo” is an online and television program, the aims of which are to analyze and clarify popular statements by the population on a given topic, in order to understand what is true and what is false. In a report published by the Centre for Research and Studies in Sociology (CIES) on the communication and disinformation regarding COVID-19 in Portugal, 71.6% of the participants said they had been exposed to misinformation; also, 32.9% of the participants said that when they identified a potentially fake piece of information, they did nothing, while 19.9% used the “CovidCheck” tool (available online: https://covidcheck.pt, accessed on 1 June 2021) that was made available in May 2020, and aimed to optimize official communication, identify misinformation and stimulate the search for secure and certified information, 16.9% confirmed the information with their family and 14.6% shared it with other people.

Most respondents to the survey agreed with the statement “washing and disinfecting hands frequently can help prevent infection with the new coronavirus”. The agreement or disagreement rates for some other statements presented to participants varied according to education level (Table 7). Regarding the statement “COVID-19 can be transmitted through food”, 30% of the respondents with secondary education said they agreed with the statement, while 43.8% of respondents with tertiary education and 50% of people with postgraduate education said they had doubts; at the same time, the majority of the respondents said they agreed with the statement “cooking food destroys the new coronavirus”.

Doubts about the possibility of SARS-CoV-2 transmission through food can be justified on the basis of news related to the presence of gastrointestinal symptoms and the detection of the virus in some frozen foods [19,21,52,53]. Thus, the main concerns of the survey participants were related to the handling of food, and the question of which food should be avoided during this period due to fear of SARS-CoV-2 infection.

The pandemic led to “new” care practices to reduce the risk of infection and transmission. Some practices were based on the idea that food and packaging could carry the virus if contaminated by sneezing, talking, or coughing without proper hygiene and food safety measures [5,6,44,45,46,95,96].

With regard to behavior during the pandemic, in the present study, the analysis was conditioned on the education variable, as the respondent’s level of education may influence the type of source consulted and the interpretation of the information, which could have an impact on behavior (Table 8). Compared to the period before the pandemic, 51.6% of respondents with a completed university (bachelor’s), technical, or professional degree said they had not changed their practices during the pandemic, while 22.8% said they had increased the frequency of certain practices compared to before the pandemic. While many of these practices may not directly prevent COVID-19, they could have had an impact on food quality, prevented foodborne illness and avoided waste; e.g., 74% and 76.7%, respectively, increased hand washing and disinfection, 39.7% paid more attention to kitchen hygiene, 37% disinfected more kitchen utensils and surfaces with bleach, 31.5% washed more fruits and vegetables under running water, 16.4% organized food in the refrigerator better to avoid cross-contamination, 12.3% checked the refrigerator temperature more frequently and 16.4% paid more attention to the expiration dates of food. The adoption of these “new” habits and behaviors may have been directly influenced by the measures disseminated by the media and social networks and supported by medical authorities, especially those related to hand washing, the use of hand sanitizer (e.g., washing and disinfecting hands before and after handling purchases) and disinfection of surfaces and the packaging of purchased products (e.g., cleaning and disinfecting packaging with soap and water, using a damp cloth and bleach diluted in water and/or 70% alcohol to destroy the outer membrane of the virus and prevent infection) [89,97,98,99,100,101,102,103,104]. These changes may also have been supported by the fact that SARS-CoV-2 was found in frozen foods and on their packaging [14,15,16,17,18,105], which may have been reflected in an increase in the consumption of home-produced foods to reduce handling, and thus the risk of contamination [54,55,58,59,60,61,62,63,64].

When considering raw and undercooked food, 48.6% said they never consumed these types of products, 20.5% said they cooked them well before eating and 6.9% said they had reduced their consumption. This could be due to fear of infection, as raw food can be handled by many people, increasing the risk of transmission, and the fact that most participants agreed that the virus is destroyed during cooking (Table 7). For example, in the US, an increase in the use of kitchen thermometers has been reported for similar reasons [61].

However, not all practices adopted can have a positive impact on food safety. For example, 32.9% increased their tendency to use all purchased food to reduce waste, and 21.9% smelled and/or tasted food more often if they were in doubt about whether it was safe to eat.

A total of 26% of participants said they were eating healthier, 17.8% said they were checking food labels more often and 11.1% said they were trying to eat in smaller portions. This is likely to have been due to changes in their routines as a result of quarantine, as well as efforts to prevent weight gain and changes in health that could have affected the functioning of the immune system. However, the National Programme for the Promotion of Healthy Eating (PNPAS) of the General Directorate of Health [106] reported an increase in the consumption of foods high in salt and sugar in the dietary habits of the Portuguese population during the pandemic. This trend can be attributed to the association of such foods with comfort, mood enhancement and stress and anxiety relief, all of which were prevalent during the pandemic [56,107,108]. Alongside this increase in unhealthy eating habits, there was also an observed rise in the consumption of healthier foods. This shift towards healthier eating choices may have been driven by the desire to improve general health, as the severity of COVID-19 had been closely linked to the presence of comorbidities, including obesity, which is directly influenced by dietary habits [57,106,107,109].

Even though the statistically significant variables did not present a strong Cramer’s V relation, it can be assumed, as observed in other studies [61,95], that behaviors related to food safety and protection against the virus were improved and that they can be influenced according to the level of education.

When professionally active people were asked about their shopping storage habits, the majority said that they washed (85%) and disinfected (63.4%) their hands before and after (73.8% and 57.3%, respectively) organizing all products. In addition, 24.1% reported that they waited a few hours before starting to organize products, 21.5% reported that they always disinfected the products’ packaging with bleach, and 19.5% did so with alcohol. Although the results were not statistically significant, there was evidence that a number of practices were in place to prevent possible infection with SARS-CoV-2 (Table 9).

During the pandemic, it was expected that the consumption and purchase of food and meals online would increase due to government restrictions aimed at containing the spread of the virus. However, this trend was not observed among respondents in Portugal, where the percentage of respondents who reported never buying food or ordering meals online remained relatively unchanged after the pandemic. Nevertheless, there was an increase in the percentage of people who reported buying food online (from 0.6% to 6.6%) and ordering meals weekly (from 4.8% to 13.3%) (Table 10). Buying food online can be seen as a cultural factor; in Brazil, the use of take-away applications increased considerably [64], a trend which was profitable for merchants. The same effect was not observed in Portugal, not only because consumers were less likely to use takeaway applications, but also because the commissions charged by these platforms were high and not profitable for Portuguese restaurants, especially those that did not have a well-established home delivery system before the pandemic. Therefore, there may not have been enough incentive to change this behavior in Portugal [110,111]. In addition, as reported by Liu et al. [112], the possibility of an increased perception that online food purchases increased the risk of infection cannot be excluded.

## 4. Conclusions

The present study showed that one of the main doubts that arose during the COVID-19 pandemic was related to the possibility of SARS-CoV-2 transmission through food, and that there was an increase in the frequency of hand washing and disinfection, disinfection of the food preparation environment, and longer cooking times due to the fear of SARS-CoV-2 infection. Despite all the information made available at national and international levels, more than half of the participants couldn’t find answers to all their questions.

The increase in the spread of fake news and increased access to this type of information do not seem to have influenced the behavior of the respondents, as most of them reported agreeing with the truths and disagreeing with the myths being disseminated.

Due to the containment measures, most of the information on various consumer issues related to the pandemic has been collected through online studies (e.g., surveys). When reviewing this information, it is important to consider the timing within the pandemic timeline and the severity of the situation experienced by the population being surveyed. This is clearly illustrated in the study by Liu et al. [112], who found that the perceived risk of buying items online was significantly higher in cities affected by the epidemic than in unaffected provinces or other regions of China. Research is now needed to assess consumer compliance with post-pandemic protective measures and the effectiveness of COVID-19 protective-measure uptake in promoting food safety practices. Understanding changes in consumer behavior and attitudes towards food safety is essential as the situation with vaccine availability continues to evolve. Further studies can provide valuable information on the long-term impact of the pandemic on food safety practices, help develop effective strategies to promote safe food handling and consumption, and ultimately prepare for a new pandemic or other emergency situation.

Consistent with the findings of this study, many other published studies have reported an increased frequency of hand and surface disinfection during the pandemic. While it is desirable for this trend to persist, it is important to examine the extent to which these practices may be excessive and unnecessary among consumers. The anxiety induced by the pandemic is widely recognized and has been associated with obsessive and compulsive washing and disinfection behaviors. It is known that some of these practices have no scientific basis. On the contrary, they can endanger the health of consumers due to the accumulation of chemical residues. For example, 27.4% of respondents in a survey in Brazil reported washing fruit with detergent [60]. Thus, it is crucial to now emphasize and debunk misconceptions surrounding these practices, as their misuse has already been shown to lead to public health and environmental issues.

### Study Limitations

Although the results showed a change in some practices due to the pandemic situation, the number of responses was low. Therefore, the results may not be representative of the Portuguese population as a whole. In addition, the fact that the survey consisted of many questions may have influenced in the participation rate. Also, as the questions were open-ended, allowing free interpretation by the respondent, it is possible that there was a lack and/or loss of information. Another limitation of the study was that the survey was conducted after the implementation and dissemination of preventive measures announced by official entities, as well as fact-checking tools, which may have influenced the results.

Due to the low response rate, it would have been interesting to assess in a larger group how the pandemic changed habits and behaviors related to food safety. However, this will have to be done a posteriori and in the context of another study, as the results of the present study were obtained in a very specific period and with many specific considerations, due to the pandemic that was being experienced.

## Figures and Tables

**Table 1:** Sociodemographic profile of the survey’s participants (*n* = 183).

	Frequency (%)
**Gender**	
Female	140 (76.5)
Male	40 (21.9)
No answer	3 (1.6)
**Age group (minimum 17; maximum 66; mean 38.3)**	
≤19	12 (6.6)
20–30	44 (24)
31–59	109 (59.6)
≥60	15 (8.2)
No answer	3 (1.6)
**Education level**	
Secondary level	40 (21.9)
Tertiary education (completed bachelor’s, technical, or professional degree)	74 (40.4)
Postgraduate education (master’s, doctorate, or post-doctoral degree)	65 (35.5)
Prefer not to say	2 (1.1)
No answer	2 (1.1)
**Location**	
Living in Portugal	168 (91.8)
Living abroad	8 (4.4) *
No answer	7 (3.8)
**Professional situation**	
Active/Employed	134 (73.2)
Not active/Not employed	47 (25.7)
No answer	2 (1.1)
**Health professional**	13 (7.1)
**Food industry professional**	35 (19.1)

* 7 in Brazil and 1 in France.

**Table 2:** Results of the association between the variable “Responsibility for shopping and/or preparing food in the household” and the sociodemographic variables. Data presented as frequency (%) *^a^*.

	Few Times	Regularly	Several Times	Always	*p* Value/Cramer’s V
Gender: Female (*n* = 140)	17 (12.1)	28 (20)	37 (26.4)	58 (41.4)	0.393/-
Age: Between 31 and 59 years (*n* = 109)	9 (8.3)	21 (19.3)	29 (26.6)	50 (45.9)	0.068/-
Level of education: Tertiary education ** (*n* = 74)	9 (12.2)	13 (17.6)	21 (28.4)	31 (41.9)	0.645/-
Location: Living in Portugal (*n* = 168)	29 (11.3)	35 (20.8)	52 (30.4)	63 (37.5)	0.394/-
Professionally active (*n* = 134)	12 (9)	22 (16.4)	43 (32.1)	57 (42.5)	0.023 */0.229
Health professional (*n* = 13)	2 (15.4)	2 (15.4)	2 (15.4)	7 (53.8)	0.578/-
Food industry professional (*n* = 35)	4 (11.4)	8 (22.8)	9 (25.7)	14 (40)	0.919/-

*^a^* Only the categories in which the largest number of responses were concentrated are presented. * The value of the Cramer’s V test is presented when there are distributions with statistically significant differences (*p* < 0.005). ** completed bachelor’s, technical, or professional degree.

**Table 3:** Results of the association between the variable “Main food and/or food safety doubts and concerns raised by the COVID-19 pandemic” and the sociodemographic variables. Data presented as frequency (%) *^a^*.

	Foods to Avoid	Food Handling	Transmission of COVID-19 through Food	How to Sanitize Reusable Bags	Other **	No Doubts	*p* Value/Cramer’s V
Gender: Female	12 (8.6)	64 (46.0)	16 (11.5)	3 (2.2)	3 (2.2)	41 (29.5)	0.043 */0.253
Age: 31–59 years	13 (11.9)	43 (39.4)	16 (14.7)	2 (1.8)	2 (1.8)	33 (30.3)	0.898/-
Level of education: Tertiary education ***	8 (10.8)	27 (36.5)	12 (16.2)	1 (1.4)	3 (4.1)	23 (31.1)	0.205/-
Location: Living in Portugal	21 (12.6)	68 (40.7)	21 (12.6)	3 (1.8)	4 (2.4)	50 (29.9)	0.098/-
Professionally active	15 (11.3)	54 (40.6)	17 (12.8)	3 (2.3)	4 (3)	40 (30.1)	0.996/-
Health professional	0 (0)	7 (53.8)	4 (30.8)	0 (0)	1 (7.7)	1 (7.7)	0.098/-
Food industry professional	3 (8.6)	12 (34.3)	7 (20)	1 (2.9)	2 (5.7)	10 (28.6)	0.611/-
Total (%)	9.1	41.6	17	1.8	3.8	26.7	

*^a^* Only the categories in which the largest number of responses were concentrated are presented. * The value of the Cramer’s V test is presented when there are distributions with statistically significant differences (*p* < 0.005); ** Other: COVID-19 transmission, efficacy of implemented measures, COVID-19 prevention; *** Completed bachelor’s, technical, or professional degree.

**Table 4:** Results of the association between the variable “Main means of communication for the clarification of doubts in relation to food and/or food safety during the COVID-19 pandemic” and the sociodemographic variables. Data presented as frequency (%) *^a^*.

	Television	Radio	Printed Newspapers and Magazines	National Directorate-General of Health (DGS) Guideline	News Websites (Portal SAPO, Notícias ao Minuto, Observador)	Official Scientific Websites	Social Network	Family and Friends	Official Health Websites	*p* Value/Cramer’s V
Gender: Female	37 (33.9)	1 (0.9)	2 (1.8)	30 (27.5)	13 (11.9)	10 (9.2)	3 (2.8)	6 (5.5)	7 (6.4)	0.709/-
Age: Between 31 and 59 years	27 (32.1)	1 (1.2)	1 (1.2)	25 (29.8)	13 (15.5)	9 (10.7)	1 (1.2)	2 (2.4)	5 (6)	0.367/-
Level of education: Tertiary education (completed bachelor’s, technical, or professional degree)	19 (31.1)	1 (1.6)	1 (1.6)	18 (29.5)	7 (11.5)	4 (6.6)	1 (1.6)	4 (6.6)	6 (9.8)	0.107/-
Location: Portugal	44 (33.3)	0 (0)	4 (3)	42 (31.8)	15 (11.4)	10 (7.6)	4 (3)	7 (5.3)	6 (4.5)	<0.001 */0.491
Professionally active	39 (36.8)	0 (0)	3 (2.8)	31 (29.2)	12 (11.3)	7 (6.6)	4 (3.8)	4 (3.8)	6 (5.7)	0.358/-
Health professional	4 (33.3)	0 (0)	0 (0)	4 (33.3)	0 (0)	1 (8.3)	1 (8.3)	0 (0)	2 (16.7)	0.602/-
Food industry professional	9 (30)	0 (0)	1 (3.3)	10 (33.3)	2 (6.7)	5 (16.7)	1 (3.3)	1 (3.3)	1 (3.3)	0.730/-
Total (%)	32.9	0.5	1.9	30.7	9.8	9.4	3.3	3.8	7.5	

*^a^* Only the categories in which the largest number of responses were concentrated are presented. * The value of the Cramer’s V test is presented when there are distributions with statistically significant differences (*p* < 0.005).

**Table 5:** Results of the association between the variable “Websites that were consulted by the survey participants to access information on food safety during the COVID-19 pandemic” and the sociodemographic variables. Data presented as frequency (%) *^a^*.

	National Directorate-General of Health (DGS)	Portuguese Food and Economic Security Authority (ASAE)	European Food Safety Authority (EFSA)	Ricardo Jorge Institute	No Website Was Consulted	*p* Value/Cramer’s V
Gender: Female	79 (58.1)	0 (0)	5 (3.7)	0 (0)	52 (38.2)	0.278/-
Age: 31–59 years	63 (60.6)	1 (1)	3 (2.9)	0 (0)	37 (35.6)	0.981/-
Level of education: Tertiary education **	41 (57.7)	1 (1.4)	2 (2.8)	0 (0)	27 (38)	0.443/-
Location: Living in Portugal	99 (60.7)	1 (0.6)	5 (3.1)	0 (0)	58 (35.6)	0.033 */0.226
Professionally active	81 (61.8)	0 (0)	3 (2.3)	0 (0)	47 (35.9)	0.106/-
Health professional	7 (53.8)	0 (0)	1 (7.7)	0 (0)	5 (38.5)	0.840/-
Food industry professional	22 (64.7)	1 (2.9)	3 (8.8)	0 (0)	8 (23.5)	0.016 */0.242
Total (%)	59.6	0.9	4.5	0	35	

*^a^* Only the categories in which the largest number of responses are concentrated were presented. * The value of the Cramer’s V test is presented when there are distributions with statistically significant differences (*p* < 0.005); ** Completed bachelor’s, technical, or professional degree.

**Table 6:** Results of the association between the variable “Survey respondents’ assessment of clarification of food and food safety doubts after consultation of information sources” and the sociodemographic variables. Data presented as frequency (%) *^a^*.

	“My Doubts Were Not Clarified Because I Did Not Look for Information.”	“I Searched, but I Did Not Find Answers to My Questions.”	“I Searched, but I Find Answers to Some of My Questions.”	“I Searched and Found Answer to All My Questions.”	*p* Value
Gender: Female	18 (14.9)	3 (2.5)	61 (50.4)	39 (32.2)	0.701
Age: 31–59 years	10 (11)	3 (3.3)	48 (52.7)	30 (33)	0.942
Level of education: Tertiary education *	10 (14.9)	1 (1.5)	33 (49.3)	23 (34.3)	0.917
Location: Living in Portugal	20 (14)	4 (2.8)	72 (50.3)	47 (32.9)	0.367
Professionally active	14 (12.3)	4 (3.5)	57 (50)	39 (34.2)	0.556
Health professional	2 (16.7)	0 (0)	7 (58.3)	3 (25)	0.784
Food industry professional	3 (9.6)	0 (0)	14 (45.2)	14 (45.2)	0.435
Total (%)	13.3	1.9	51	33.8	

*^a^* Only the categories in which the largest number of responses were concentrated are presented. * Completed bachelor’s, technical, or professional degree.

**Table 7:** Opinion on myths and truths related to COVID-19 according to level of education. Data presented as frequency (%).

	Secondary Education				** Tertiary Education				*** Postgraduate Education				*p* Value/Cramer’s V
	Agree	I Have Doubts	Disagree	I Do not Have an Opinion	Agree	I Have Doubts	Disagree	I Do not Have an Opinion	Agree	I Have Doubts	Disagree	I Do not Have an Opinion	
Eating garlic can help prevent infection with the new coronavirus.	2 (5)	3 (7.5)	23 (57.5)	12 (30)	5 (5.8)	12 (16.4)	41 (56.2)	15 (20.5)	0 (0)	10 (15.4)	45 (69.2)	10 (15.4)	0.149/-
Adding pepper to soup can help prevent infection with the new coronavirus.	1 (2.5)	3 (7.5)	24 (60)	12 (30)	0 (0)	9 (12.3)	47 (64.4)	17 (23.3)	0 (0)	3 (4.6)	53 (81.5)	8 (13.8)	0.079/-
Drinking alcohol can help prevent infection with the new coronavirus.	2 (5)	2 (5)	27 (67.5)	9 (22.5)	1 (1.4)	7 (9.6)	58 (79.5)	7 (9.6)	0 (0)	3 (4.6)	57 (87.7)	5 (7.7)	0.071/-
Drinking water with a few drops of bleach can prevent infection with the new coronavirus.	1 (2.5)	2 (5)	30 (75)	7 (17.5)	0 (0)	4 (5.5)	65 (89)	4 (5.5)	0 (0)	3 (4.6)	60 (92.3)	2 (3.1)	0.061/-
Eating citrus fruits (e.g: orange, lemon) can help prevent infection with the new coronavirus.	5 (12.5)	10 (25)	15 (37.5)	10 (25)	16 (21.9)	22 (30.1)	24 (32.9)	11 (15.1)	9 (14.1)	15 (23.4)	31 (48.4)	9 (14.1)	0.359/-
Soaking fruits and vegetables in lemon water can help prevent infection with the new coronavirus.	4 (10)	10 (25)	14 (35)	12 (30)	6 (8.2)	23 (31.5)	29 (39.7)	15 (20.5)	5 (7.7)	21 (32.3)	30 (46.2)	9 (13.8)	0.589/-
Soaking fruits and vegetables in vinegar can help prevent infection with the new coronavirus.	9 (23.1)	6 (15.4)	12 (30.8)	12 (30.8)	9 (12.5)	21 (33.3)	27 (37.5)	12 (16.7)	8 (12.3)	24 (36.9)	27 (41.5)	6 (9.2)	0.033*/0.197
Washing and disinfecting the hands frequently helps prevent infection with the new coronavirus.	35 (87.5)	0 (0)	1 (2.5)	4 (10)	66 (90.4)	4 (5.5)	2 (2.7)	1 (1.4)	62 (95.4)	1 (1.5)	1 (1.5)	1 (1.5)	0.105/-
Drying hands in the dryer eliminates the new coronavirus.	1 (2.5)	4 (10)	25 (62.5)	10 (25)	2 (2.7)	17 (23.3)	49 (67.1)	5 (6.8)	0 (0)	9 (14.1)	50 (78.1)	5 (7.8)	0.024 */0.202
COVID-19 can be transmitted through food.	12 (30)	7 (17.5)	10 (25)	11 (27.5)	9 (12.3)	32 (43.8)	19 (26)	13 (17.8)	7 (10.9)	32 (50)	18 (28.1)	7 (10.9)	0.008 */0.222
Cook food well destroys the new coronavirus.	16 (41)	6 (15.4)	6 (15.4)	11 (28.2)	39 (53.4)	17 (23.3)	11 (15.1)	6 (8.2)	42 (64.6)	11 (16.9)	3 (4.6)	9 (13.8)	0.023 */0.203
COVID-19 can be transmitted through flies and other insects.	4 (10)	6 (15)	14 (35)	16 (40)	2 (2.7)	30 (41.1)	26 (35.6)	15 (20.5)	1 (1.6)	14 (21.9)	33 (51.6)	16 (25)	0.004 */0.327

* The value of the Cramer’s V test is presented when there are distributions with statistically significant differences (*p* < 0.005); ** completed bachelor’s, technical, or professional degree; *** master’s, doctorate, or post-doctoral degree.

**Table 8:** Food handling and consumption practices during the COVID-19 pandemic in comparison with previous situation, by education level “Tertiary education (completed bachelor’s, technical, or professional degree)”. Data presented as frequency (%).

	Never	Less	No Change	More	*p* Value/Cramer’s V
Before shopping, I check what I have at home and plan my purchases according to it	0 (0)	2 (2.7)	44 (60.3)	27 (37)	0.270/-
At home, I pay attention to the expiration date of foods.	1 (1.4)	1 (1.4)	59 (80.8)	12 (16.4)	0.030 */0.198
I try to use all foods and reduce the amount of waste.	0 (0)	0 (0)	49 (67.1)	24 (32.9)	0.012 */0.223
My diet is not varied.	15 (20.8)	9 (12.5)	41 (56.9)	7 (9.7)	0.923/-
I prepare meals at home.	0 (0)	1 (1.4)	40 (54.8)	32 (43.8)	0.109/-
I have a healthy diet.	1 (1.4)	5 (6.8)	48 (65.8)	19 (26)	0.447/-
I use food supplements, such as vitamins	35 (49.3)	6 (8.5)	22 (31)	8 (11.3)	0.086/-
I try to eat in small portions.	10 (13.9)	8 (11.1)	46 (63.9)	8 (11.1)	0.009 */0.220
I pay attention to kitchen hygiene, keeping clean utensils and surfaces.	0 (0)	1 (1.4)	43 (58.9)	29 (39.7)	0.755/-
I disinfect kitchen utensils and surfaces with bleach.	18 (24.7)	3 (4.1)	25 (34.2)	27 (37)	0.005 */0.230
I often wash my hands.	0 (0)	1 (1.4)	18 (24.7)	54 (74)	0.680/-
I often disinfect my hands.	1 (1.4)	2 (2.7)	14 (19.2)	56 (76.7)	0.365/-
I cook food well.	1 (1.4)	0 (0)	57 (78.1)	15 (20.5)	0.002 */0.221
I wash fruits and vegetables carefully with running water.	0 (0)	2 (2.7)	48 (65.8)	23 (31.5)	0.028 */0.175
I disinfect fruits and vegetables with vinegar.	29 (39.7)	6 (8.2)	24 (32.9)	14 (19.2)	0.530/-
I disinfect fruits and vegetables with lemon.	43 (58.9)	6 (8.2)	21 (28.8)	3 (4.1)	0.332/-
I disinfect fruits and vegetables with bleach.	45 (62.5)	3 (4.2)	15 (20.8)	4 (10.5)	0.559/-
I disinfect fruits and vegetables with an appropriate product that I buy.	51 (69.9)	3 (4.1)	13 (17.8)	6 (8.2)	0.432/-
If I am sick, I do not cook for my family.	14 (20.6)	3 (4.4)	37 (54.4)	14 (20.6)	0.314/-
I carefully check food labels.	4 (5.5)	5 (6.8)	51 (69.9)	13 (17.8)	<0.001 */0.257
I eat backyard eggs.	22 (30.1)	7 (9.6)	38 (52.1)	6 (8.2)	0.139/-
I consume vegetables from my Garden or from small producers.	21 (28.8)	2 (2.7)	32 (43.8)	18 (24.7)	0.062/-
I consume meat from animals raised by me or by small producers.	41 (56.9)	4 (5.6)	22 (30.6)	5 (6.9)	0.514/-
I eat undercooked/underdone foods (eggs, meat, fish).	35 (48.6)	5 (6.9)	30 (41.7)	2 (2.8)	0.062/-
I do not eat food beyond the expiration date.	14 (19.2)	7 (9.6)	49 (67.1)	3 (4.1)	0.009 */0.219
I store food in the fridge in a way to prevent cross-contamination.	7 (9.6)	3 (4.1)	51 (69.9)	12 (16.4)	0.046 */0.191
I store eggs in the fridge.	4 (5.5)	4 (5.5)	53 (72.6)	12 (16.4)	0.056/-
I smell and/or taste foods when I have doubts whether it is fit for consumption.	5 (6.8)	3 (4.1)	49 (67.1)	16 (21.9)	0.066/-
I check fridge temperature.	14 (19.2)	2 (2.7)	48 (65.8)	9 (12.3)	0.006 */0.226
Total (%)	20.6	5	51.6	22.8	

* The value of the Cramer’s V test is presented when there are distributions with statistically significant differences (*p* < 0.005).

**Table 9:** Organization of purchases during the COVID-19 pandemic by professionally active people. Data presented as frequency (%).

	Never	Sometimes	Always	Does Not Apply	*p* Value
I wash my hands before organizing purchases.	5 (3.8)	15 (11.3)	113 (85)	0 (0)	0.714
I disinfect my hands before organizing purchases.	22 (16.8)	23 (17.6)	83 (63.4)	3 (2.3)	0.755
I use gloves to organize purchases.	114 (87)	7 (5.3)	6 (4.6)	4 (3.1)	0.221
I do not store food right away.	53 (39.8)	45 (33.8)	25 (18.8)	10 (7.5)	0.224
I do not touch food for at least a few hours.	70 (52.6)	32 (24.1)	17 (12.8)	14 (10.5)	0.834
I do not touch food for at least one day.	88 (67.2)	20 (15.3)	7 (5.3)	16 (12.2)	0.580
I do not touch food for at least for 72 h.	95 (72)	10 (7.6)	11 (8.3)	16 (12.1)	0.095
I clean the packages of the products I bought with a damp cloth.	74 (56.1)	25 (18.9)	26 (19.7)	7 (5.3)	0.807
I clean the packages of the products I bought with bleach.	80 (61.5)	16 (12.3)	28 (21.5)	6 (4.6)	0.223
I clean the packages of the products I bought with alcohol.	68 (53.1)	26 (20.3)	25 (19.5)	9 (7)	0.337
I wash my hands after organizing purchases.	7 (5.4)	26 (20)	96 (73.8)	1 (0.8)	0.670
I disinfect my hands after organizing purchases.	23 (17.6)	29 (22.1)	75 (57.3)	4 (3.1)	0.627

**Table 10:** Online purchase of food and meals before and during the COVID-19 pandemic among residents in Portugal. Data presented as frequency (%).

	Never	Less than Once a Month	Once a Month	Weekly	*p* Value/Cramer’s V
Online food purchase before the pandemic	137 (82.5)	19 (11.4)	9 (5.4)	1 (0.6)	0.019 */0.238
Online meals purchase before the pandemic	97 (58.4)	40 (24.1)	21 (12.7)	8 (4.8)	0.003 */0.284
Online food purchase during the pandemic	121 (72.9)	33 (19.9)	0 (0)	11 (6.6)	0.020 */0.238
Online meals purchase during the pandemic	96 (57.8)	44 (26.5)	0 (0)	22 (13.3)	0.001 */0.304

* Statistically significant results (*p* < 0.05).

## Data Availability

All data are available in this manuscript.

## Supplementary Materials

The following supporting information can be downloaded at: https://www.mdpi.com/article/10.3390/foods12142802/s1, Supplementary Information.
