# Supplementary material for: Access to Information, and Concerns, Myths and Truths about Food Safety during the COVID-19 Pandemic: An Overview of the Portuguese Population
Source: Foods. 2023 Jul 24;12(14):2802. doi: 10.3390/foods12142802 (PMC10380000; doi:10.3390/foods12142802)
Supplement: Supplementary file 1 [file foods-12-02802-s001.zip › foods-2491456-supplementary.pdf]

Survey translated from portuguese to English

<u>Food safety during COVID-19 pandemic</u>

The answers to be collected will allow us to know and assess habits and behaviors related to the handling and consumption of food that may have been altered by the COVID-19 pandemic. The survey is anonymous and will be used exclusively by researchers from the Superior School of Biotechnology of Catholic University from Porto, Portugal.

1. I accept the above statement and intent to continue the survey.
   Yes ( ) No ( )
2. Are you responsible for the purchase and/ or preparation of food in the household?
   Never ( ) Few times ( ) Regularly ( )
   Several times ( ) Always ( )
   If you answered "never" to question 2, the survey ends.
3. During the pandemic, have you had doubts about food-related issues, for example (check only the cases in which you had doubts):
   ( ) Foods to avoid
   ( ) How to wash foods
   ( ) How to disinfect foods
   ( ) How to cook food
   ( ) If COVID-19 could be transmitted through food
   ( ) How to sanitize packages (cans, bottles, cuvettes) after purchase
   ( ) How to sanitize reusable bags after purchase
   ( ) Other: ______________________________
   ( ) I had no doubt
4. In case of doubts, how they were clarified?
   ( ) Television
   ( ) Radio
   ( ) Printed magazines and newspapers
   ( ) National Directorate-General of Health (DGS) guideline
   ( ) News websites (such as Portal SAPO, Observador, Notícias ao Minuto)
   ( ) Official entities websites, such as universities
   ( ) Social network
   ( ) Family and friends
   ( ) Official health entities
5. Have you consulted any of the following websites to access information on food safety during COVID-19 pandemic?
   ( ) National Directorate-General of Health (DGS)
   ( ) Portuguese Food and Economic Security Authority (ASAE) (https://www.asae.gov.pt/covid-19-asae.aspx)
   ( ) European Food Safety Authority (EFSA) (https://www.efsa.europa.eu/en/news/coronavirus-no-evidence-food-source-or-transmission-route)
   ( ) Ricardo Jorge Institute (http://www.insa.pt/)
   ( ) No website was consulted

6. Have you got the necessary clarification of doubts?
( ) My doubts were not clarified because I did not look for information
( ) I searched, but I did not find answers to my questions
( ) I searched, but I find answers to some of my questions
( ) I searcher and found answers to all my questions
7. How the following statements describe your behavior during COVID-19 pandemic compared to the previous situation:

	Never	Less	No change	More
Before shopping, I check what I have at home and plan my purchases according to it				
At home, I pay attention to the expiration date of foods				
I try to use all foods and reduce the amount of waste				
My diet is not varied				
I prepare meals at home				
I have a healthy diet				
I use food supplements, such as vitamins				
I try to eat in small portions				
I pay attention to kitchen hygiene, keeping clean utensils and surfaces				
I disinfect kitchen utensils and surfaces with bleach				
I often wash my hands				
I often disinfect my hands				
I cook food well				
I wash fruits and vegetables carefully with running water				
I disinfect fruits and vegetables with vinegar				
I disinfect fruits and vegetables with lemon				
I disinfect fruits and vegetables with bleach				
I disinfect fruits and vegetables with an appropriate product that I buy				
If I am sick, I do not cook for my family				
I carefully check food labels				
I eat backyard eggs				
I consume vegetables from my Garden or from small producers				
I consume meat from animals raised by me or by small producers				
I eat undercooked/ underdone foods (eggs, meat, fish)				
I do not eat food beyond the expiration date				
I store food in the fridge in a way to prevent cross-contamination				
I store eggs in the fridge				
I smell and/ or taste foods when I have doubts whether it is fit for consumption				
I check fridge temperature				

8. Do you used to buy food or meals online before the pandemic?

	Never	Less than once a month	Once a month	Weekly	More than once a week
Purchase of food online					
Purchase of meals online					

9. Do you buy food or meals online during the pandemic?

	Never	Less than once a month	Once a month	Weekly	More than once a week
Purchase of food online					
Purchase of meals online					

10. During the pandemic, when you do shopping, how do you do your storage?

	Never	Sometimes	Always	Does not apply
I wash my hands before organizing purchases				
I disinfect my hands before organizing purchases				
I use gloves to organize purchases				
I do not store food right away				
I do not touch food for at least a few hours				
I do not touch food for at least one day				
I do not touch food for at least for 72 hours				
I clean the packages of the products I bought with a damp cloth				
I clean the packages of the products I bought with bleach				
I clean the packages of the products I bought with alcohol				
I wash my hands after organizing purchases				
I disinfect my hands after organizing purchases				

11. What is your opinion regarding the following statements:

	Agree	I have doubts	Disagree	I do not have an opinion
Eating garlic can help prevent infection with the new coronavirus				
Adding pepper to soup can help prevent infection with the new coronavirus				
Drinking alcohol can help prevent infection with the new coronavirus				
Drinking water with a few drops of bleach can prevent infection with the new coronavirus				
Eating citrus fruits (for example: orange, lemon) can help prevent infection with the new coronavirus				

Soaking fruits and vegetables in lemon water can help prevent infection with the new coronavirus				
Soaking fruits and vegetables in vinegar can help prevent infection with the new coronavirus				
Washing and disinfecting the hands frequently helps prevent infection with the new coronavirus				
Drying hands in the dryer eliminates the new coronavirus				
COVID-19 can be transmitted through food				
Cook food well destroys the new coronavirus				
COVID-19 can be transmitted through flies and other insects				

12. What is your gender?

    Female ( )   Male ( )

13. What is your age? ____________
14. Place of residence (city/ country): ________________________
15. What is the higher educational level completed?
    - ( ) No level of complete education
    - ( ) Secondary education level
    - ( ) Tertiary education level (completed bachelor, technical or professional degree)
    - ( ) Postgraduate education (master, doctorate, or post-doctorate degree)
    - ( ) I prefer not to say
16. What is your current employment situation?
    - ( ) Professionally active/ employed (full or part-time)
    - ( ) Not professionally active (unemployed, students with unpaid working experience, retired, permanently disabled and/or incapable of performing any tasks)
17. Do you work or used to work as a health professional?

    Yes ( )   No ( )

18. Do you work or used to work as a food-industry professional?

    Yes ( )   No ( )
